# Eating disorders in the Arab world: a literature review

**DOI:** 10.1186/s40337-020-00336-x

**Published:** 2020-11-06

**Authors:** Bernou Melisse, Edwin de Beurs, Eric F. van Furth

**Affiliations:** 1 Novarum Center for Eating Disorders & Obesity, Jacob Obrechtstraat 92, 1071 KR Amsterdam, the Netherlands; 2 Rivierduinen Eating Disorders Ursula, Sandifortdreef 19, 2333 ZZ Leiden, Netherlands; 3 Department of Psychiatry, Leiden University Medical Center, Albinusdreef 2, 2333 ZA Leiden, Netherlands; 4 Arkin Mental Health Institute, research department, Klaprozenweg 111, 1033 NN Amsterdam, the Netherlands; 5 Leiden University, Section Clinical Psychology, Wassenaarseweg 52, 2333 AK Leiden, the Netherlands

**Keywords:** Arab, Eating disorders, Prevalence, Eating disorder-related variables, Desire to be thin, Correlates

## Abstract

**Background:**

The prevalence of eating disorders has been assumed to be low in the Arab world, due to the alleged absence of the thin ideal. However, the Arab world is undergoing rapid sociocultural changes, and there are reports of an increase of the desire to be thin. This literature review therefore provides point-prevalence of Arabs at high risk for eating disorders, and a comprehensive synthesis of correlates of eating disorder symptoms, eating disorder-related variables and of a high risk for eating disorders.

**Method:**

Several electronic databases were searched for published, peer-reviewed studies between 1986 and 2019 involving several key terms. From 317 screened studies, 81, mainly cross-sectional, were included. Preferred Reporting Items for Systematic reviews and meta-analyses was used as guidance and the quality of studies were assessed using the Newcastle-Ottawa scale.

**Results:**

Estimates of individuals at high risk for eating disorders ranged from 2 to 54.8%. The eating disorder-related variables identified were desire to be thin, body dissatisfaction, disturbed-, and dieting- eating behavior. Identified correlates were increased affluence, media use, western influences, and obesity. An additional finding was that in some cases eating disorders were expressed somatically rather than psychiatrically.

**Discussion:**

In the Arab world, females were most at risk for eating disorders and eating disorder symptoms. Sociocultural changes gave rise to the thin ideal and the prevalence of obesity, increasing the risk for the development of eating disorder-related variables and eating disorders. The literature on eating disorders in the Arab world suffers from potential limitations due to the use of non-validated assessment tools. Further research is necessary, particularly on the development and validation of a culturally sensitive assessment tool. Improved knowledge is likely to increase the number of people seeking treatment and decrease the stigma of psychotherapy.

## Plain English summary

In the Arab world a curvy body was perceived as fertile, so eating disorders were assumed to be rare. In recent years, however, due to rapid sociocultural changes, Western influences, media use, and increased affluence, Arabs have started to admire a thinner body. Consequently, research has now begun to address the existence of eating disorders in the Arab world. The increased popularity of the desire to be thin has been associated with soft symptoms such as disturbed eating behavior, body dissatisfaction and dieting behavior. In addition to Arabs at risk for eating disorders, eating disorder symptoms identified include binge eating, self- induced vomiting, and laxative use. Sometimes, eating disorders manifest themselves in a different way in the Arab world: some Arabs have expressed eating disorders somatically, as nausea, stomach ache, and so on, rather than psychiatrically. There is a need for assessment tools to be adjusted to the Arab culture. Improved knowledge will facilitate recognition of eating disorders, encourage people to seek treatment and decrease the stigma of psychotherapy.

## Background

Eating disorders (EDs) have a significant impact on the well-being of affected individuals [1]. This includes comorbid obesity [2], depressive symptoms, anxiety [3] substance abuse, suicide attempts [4], and high rates of mortality and relapse [5]. Since EDs have historically been associated with Caucasian females in developed Western countries with high socio-economic status [6], they have been perceived as culturally bound syndromes [7]. Taking this in consideration most studies regarding EDs have been heavily concentrated in Western countries [8].

Cultural factors are essential to understanding the development of EDs [9] and the main feature identified associated with EDs in the West is a thin body ideal [6], which is presented to the society as achievable by dieting and exercising [8]. Traditional Arab notions of beauty are different, with the curvy body ideal associated with fertility and wealth [10] EDs were assumed not to afflict Arabs^1^ [10, 11] hence EDs were not reported in the Arab world until 1986 [12]. However, from then on [10, 11] reports on the thin ideal in the Arab world steadily increased [13–15].

Recent studies have shown that EDs occur globally [16, 17], EDs occur particularly in cultures in transition as they adopt Western values [7, 18], illuminating the interplay between culture and psychopathology [19]. This is relevant to the Arab world, since the oil boom of the 1970s and the consequent increased affluence [20, 21], it has been undergoing rapid sociocultural and socioeconomic changes [14, 22]. The oil boom led to the arrival of Western companies, Western expatriates [18, 23], and to increased exposure to Western culture [8].

The sociocultural changes associated with acculturation include adopting the language, lifestyle, values, and beliefs of other cultures [24]. In the Arab world, increased exposure to Western media [20, 25–27], and increased contact with expatriates [28, 29] has also led to a rise in the popularity of the thin ideal [10, 30], and to increased levels of dieting, body dissatisfaction and EDs [8]. However, the theory of Westernization assumes that the Western culture is transferrable to Arab cultures [31], the oil boom has also been associated with elevated levels of industrialization [18] including increased technology, affluence, and higher education [32, 33]. Industrialization in particular has coincided with changes in the types of food available [7, 34], and these are instrumental in a rise in obesity [28, 35, 36]. Together, these changes have contributed to elevated levels of non-communicable diseases such as diabetes mellitus, hypertension [20], and psychological problems [37] including ED-related variables and EDs [18, 38–40].

Kraemer and colleagues [41–43] have provided us with a terminology to address how culture and EDs may be associated. An important distinction is that between a correlate and a risk factor. A correlate is a potential risk factor that is measured at the same time as an ED. The correlate and ED are associated with each other, but precedence of the correlate to the onset of an ED is not proven [41, 42]. Therefore correlates cannot be defined as risk factors. Correlates are identified in cross sectional studies [41–43]. Currently identified correlates are increased affluence [6, 43], obesity [44, 45], and societal changes such as industrialization [46], globalization [6], and acculturation [38, 46]. If it is established that an associated variable precedes the onset of an ED, this variable may be indicated as a risk factor [42]: gender is an example [41]. A risk factor identifies individuals who are at elevated risk for future emergence of an ED [43], and risk factors can best be identified by longitudinal studies [41, 42]. Initiation of the onset of an ED can be indicated by ED-related variables [43]. ED-related variables are an early sign of an ED which indicate vulnerability to develop an ED. However, on their own ED-related variables do not meet the criteria for an ED as described in the DSM-5 [47]. Examples of ED-related variables are disturbed eating behavior, self-reported dieting, an elevated desire to be thin, and body dissatisfaction [41].

To distinguish between various levels of ED-pathology, commonly the following types are described: ED symptoms, at high risk for ED and full syndrome ED [41–43]. ED symptoms are symptoms that are defining features of an ED [43] such as binge eating, and compensatory behaviors, which can be measured by self-report and clinical interviews. If the frequency of ED symptoms meets the criteria of an ED, including clinically significant functional impairment [6], an individual can be classified as suffering from a full syndrome ED [43, 47]. Full syndrome EDs are best diagnosed with a clinical interview [43]. Individuals who score above a clinical cut-off on an ED-screening instrument are considered to be at high risk for an ED [43, 48].

This review will differentiate between ED-related variables, ED symptoms, and high risk for EDs. Arabs with elevated levels of ED-related variables and ED symptoms are at risk for EDs, as are Arabs who score above a clinical cut-off on an ED-screening instrument, who will be referred to as Arabs at high risk for EDs. Correlates of ED-related variables, ED symptoms, and scores above a clinical cut-off on an ED-screening instrument will also be identified. Identification of correlates and ED-related variables is likely to facilitate identification of risk groups for prevention programs [43, 49]. At the same time, preventative and treatment programs cannot be copied from Western societies [50]. They need to be adapted to the relevant sociocultural context [51, 52].

Although EDs have been reported in the Arab world [28, 29, 36, 53–55], there is limited valid data [56], and the substantial differences in the expression of EDs among different cultures [7, 52, 57, 58] are yet to be taken into account. For example, somatic expression of EDs is more likely in the Arab world than in the West [52, 58]. Some Arabs attribute a restrictive food pattern to somatic complaints [59], while there are also reports of body dissatisfaction and fear of fatness [52, 58]. Examples of somatic complaints are stomach ache, feeling bloated or an absence of appetite [59]. In addition, perceptions of body image also differ between cultures [52].

There are a number of comprehensive reviews of the nature of EDs in Western countries [5, 43, 51, 60, 61]. However, as the Arab world has undergone rapid sociocultural changes [14, 22], EDs could be more culturally reactive than culturally bound [8]. An overview of EDs in the Arab world is needed. Unfortunately, due to a lack of appropriate and valid data, it is not feasible to examine how the prevalence of EDs in the Arab world has changed over the years. Still, data from ED-screening instruments can help identify individuals at high risk for EDs, and enable examination of associations between ED-related variables, correlates and ED symptoms. In this review, we therefore aim to provide estimates regarding the point-prevalence of Arabs at high risk for EDs, with ED symptoms above threshold values for caseness, and to provide a comprehensive synthesis of relevant studies of correlates and ED-related variables. The countries included here are those together referred to as “the Arab world”, which is a part of the Eastern Mediterranean Region [62], also referred to as the Middle East [63]. Non-Arab countries such as Cyprus and Turkey are excluded [64]. To the best of our knowledge, this is the first review summarizing EDs in the Arab world.

## Method

### Search strategy

**Fig. 1:**
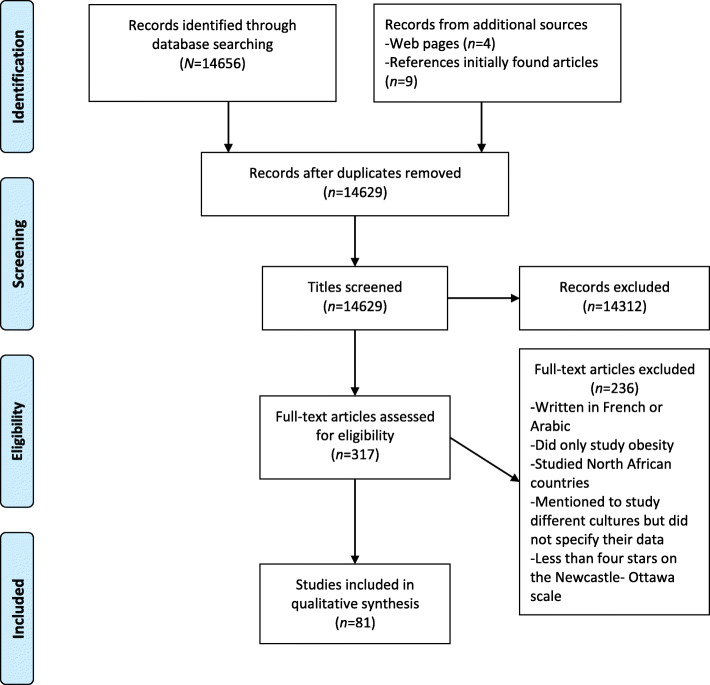
Flow chart of study selection process based on PRISMA

The primary search was conducted in Web of Science, PubMed (Medline) and Google Scholar databases from 1986 up to July 2019 by BM, and involved key terms related to ED prevalence in the Arab world. All combinations of (“Eating disorders” OR “Anorexia Nervosa” OR “Bulimia Nervosa” OR “Binge Eating Disorder” OR “Disturbed Eating Behavior” OR “Eating Attitudes” OR “Dieting” OR “Body Image” OR” Body Satisfaction” OR “Obesity”) AND (“Arab” OR “Middle East” OR “Gulf” OR “United Arab Emirates (UAE)” OR “Saudi” OR “Oman” OR “Qatar” OR “Bahrain” OR “Kuwait” OR “Lebanon” OR “Palestine” OR “Jordan”) were searched. To be included, articles had to be peer reviewed. This resulted in 14,656 hits. Subsequently, more records were added from other sources (*n* = 13). After duplicates were removed, 14,629 studies remained. The titles were then screened for eligibility. Eligible studies were conducted in Arab countries in the Middle East, providing estimates regarding the point prevalence of individuals at high risk for EDs, ED symptoms and ED-related variables, and reporting correlates of scores above a clinical cut-off in ED-screening instruments, ED symptoms, and ED-related variables. Records (*n* = 14,312) studying, for example, diabetes mellitus, metabolic syndrome or nutrition related to other diseases, were excluded. When there was doubt, the abstract and discussion section were further screened. This resulted in 72 relevant studies, plus 9 additional studies identified from their lists of reference. The additional sources of studies were four websites involving statistical data such as GCC stat (Fig. 1). Most studies were conducted in the UAE (*n* = 11), followed by Jordan and Saudi Arabia (*n* = 7). A further six studies were conducted in Kuwait, five in Egypt, four each in Oman, and Qatar. Three among Palestinian populations, two each in Bahrain, Lebanon, Iran, and one each in Algeria, and Libya.

### Inclusion criteria

Inclusion criteria were that the studies involved Arab Middle Eastern countries [63] of the Eastern Mediterranean Region [62]. These are West Asian countries (Lebanon, Jordan, Iran), the Gulf (Bahrain, Kuwait, Qatar, UAE, Saudi Arabia, Oman), Egypt, and Palestinian populations. Though, a non-Arab country, Iran is included because of the several cultural similarities (e.g. hijab and full body coverage for females in public) [64]. All studies investigated at least one ED symptom or ED-related variable. In cases where obesity was studied, study of at least one additional ED behavior was required. There were no restrictions on age or gender. All studies reported point prevalence, the prevalence at a certain point in time [51]. Additional studies examined correlates of EDs, ED symptoms or ED-related variables.

### Exclusion criteria

Studies involving non-Middle Eastern countries, and non-Arab Middle Eastern countries were excluded [63], as were studies focusing only on obesity, and qualitative studies without empirical data.

### Assessments

While correlates and ED-related variables can be accurately measured by self-report [43], diagnostic interviews are preferable for ED symptoms. Since diagnostic concordance between diagnostic interviews and self-report is moderate at best [65], and EDs can best be assessed by diagnostic interviews [43], questionnaires including the SCOFF, EAT, and EDE-Q may at best provide prevalence estimates of Arabs at high risk for EDs, indicated by elevated scores at ED-screening instruments. However, since for several Arab populations norms of ED-screening instruments were not available, norms of either Western populations or other Arab populations were used. Therefore, these instruments indicated elevated scores on ED-screening instruments in the target country using the norms of another country. In this study, Arabs who scored above a clinical cut-off are referred to as Arabs at high risk for EDs, regardless of the norms used.

### Data abstraction and quality assessment

This review is based on studies published in English between 1986 and 2019; a large timeframe was chosen due to the limited number of studies on EDs conducted in the Arab world. Since only studies on point prevalence were found and none on incidence rate and lifetime prevalence, prevalence refers to point prevalence. Most of the studies were published in peer reviewed journals. Two theses were also included [66, 67]. In total, 81 studies met the inclusion criteria. Almost all the studies were quantitative; most were cross sectional, although three were case control studies [35, 68, 69]. One validation study was included [54], and longitudinal data were not available. Eight cross sectional studies involved cross cultural aspects [11, 23, 26, 39, 52, 58, 64, 70]. Author BM assessed the risk of bias on study validity. Discrepancies or uncertainties were resolved by discussion with either the second or the third author. Data extraction was done independently by BM, taking into consideration study characteristics, demographics, study results, and key study outcomes. Quality of included studies was assessed using the Newcastle-Ottawa scale [71], a seven item scale that investigates power, research design, sample, recruitment, and statistical analysis (see supplementary material). The maximum score was 10 stars, based on selection (maximum five stars), comparability (maximum two stars) and outcome (maximum three stars). Only studies with at least four stars were included in the review. Preferred Reporting Items for Systematic reviews and Meta-Analyses (PRISMA) was used as guidance (Fig. 1) [72].

## Results

### Quality assessment

Of 317 studies assessed for eligibility, 81 studies met the minimum criteria of four stars on the Newcastle Ottawa scale (Fig. 1) [71]. However, the 81 studies included had several limitations, these studies either involved a selected group of users or had a selective sample. For example, the association between media and EDs in Kuwait was assessed only on female adolescents at one university [73], disturbed eating behavior only amongst adolescent girls in the UAE, and only among the inhabitants of al Ain province [20], and in Saudi Arabia, only in the city of Hail [67], all involving academically inclined schoolgirls [39]. Most of the studies involved a population of university students [74]. In addition, reporting in some studies was incomplete (lack of demographic data, such as mean age, and marital status) [67, 75]. In other studies mean and SD [26, 29, 39, 54, 55, 64, 76–79] or percentage that scored above the clinical cut-off [52, 58, 64] on the ED-screening instrument were not reported.

Another limitation was that power calculations were not reported in some studies, so their sample size was not justified and was low. This was the case (*N*=100) in an Omani [29], a Saudi [67], and (*N*=104) an Iranian [64] sample. Studies examining the association between Westernization and ED had only small samples (*N*<100) in the UAE [40], and in Iran [64].Another limitation was that several studies used non-validated ED-screening instruments. Several studies, for instance, translated the EAT-40 without adaptations, and used Western norms [20, 28, 39]. In another study on ED in Saudi Arabia, the EAT-26 was translated without adaptations, and applied using Western norms [67]. The Saudi version of the EAT-26, which included the Western norms was also used in non-Saudi countries [14, 18, 26, 40, 52, 53, 58, 74, 79, 80]. Other studies investigated adults using a version of the Saudi EAT-26 validated in a high school population [26]. Musaiger and Al-Mannai [73] also examined a Kuwaiti sample using questions validated in a Bahraini sample. In addition, two studies assessed disturbed eating behavior among adolescent Omani and European expatriates residing in Oman using the EAT26 and the EDI-2. These studies found that Omanis had significant higher EAT26 scores, while European expatriates had significantly higher EDI-2 scores. Omanis and European expatriates scored significantly different on both ED-screening instruments [52, 58], possibly due to the fact that a Saudi version including Saudi norms were used for the EAT26 and EDI-2 [78, 81]. Several studies also failed to provide essential information on the norms used, and some studies provided no information at all. One study, in Kuwait [75], did not provide information about how their statistical analysis was conducted. Furthermore, none of the studies on binge eating behavior mentioned the frequency of binge eating episodes, or whether the binges were of a subjective or objective nature [29, 70, 74, 77]. All the studies discussed relied on self-report measures, which are prone to socially desirable responding [75]. The collectivistic culture of the Arab world makes people hesitant to report individual desires [76], so the use of self-report measures makes studies vulnerable to under- or overestimation of complaints [77].

In conclusion, most of the studies included in this synthesis have limitations. These include the absence of psychometric validation of assessment tools for the Arab world, and the absence of necessary data such as demographic data, power calculation proper assessment, and description of ED-pathology.

### Eating disorder-related variables

Table 1 presents a summary of 19 studies including ED-related variables. These were: desire to be thin, body dissatisfaction, disturbed eating behavior, and dieting [43]. Disturbed eating behavior was involved in nine studies, body dissatisfaction in ten studies, desire to be thin in seven, and dieting in five cross sectional studies. ED-related variables were identified by self-report in all studies, except for one, which used both interview ánd self- report data [39]. All ED-related variables were mainly assessed in female adolescents.

**Table 1:** Summary of studies reporting eating disorder-related variables in the Arab world

Country/ Population	Authors (year)	Participants	Study design	Measures	Eating disorder-related variables	***M*** (***SD***)/OR [95% CI]/ ***r***
Algeria, Jordan, Kuwait, Libya, Palestinians residing in al-Khalil, Syria, UAE	Musaiger et al., (2013) [26]	*N* = 4698, 2240 male, 2458 female, age 15–18	Cross sectional	EAT 26	Disturbed eating behavior: twice as high in females than in males in Jordan, Libya, Palestinians residing in al-Khalil and Syria	Disturbed eating behavior: (*p* < 0.000), males as reference Jordan OR 2.96 [2.19–4.01] Libya OR 2.02 [1.37–2.98] Palestinians residing in al-Khalil OR 2.11 [1.39–3.22] Syria OR 2.75 [2.02–3.77]
Bahrain	Al-Sendi, Shetty, & Musaiger (2004) [82]	*N* = 504, 249 male, 257 female, age 12–17	Cross sectional	FRS (self- developed)	Body dissatisfaction: female: 50%, male: 30%	not reported
Egypt	Ford, Dolan, & Evans (1990) [10]	*N* = 218, 61 male, mean age = 20.0, 169 female, mean age = 19.5, university students	Cross sectional	FRS (self- developed)	Thin ideal: female: ideal shape significantly thinner than their actual shape; male & female: preference for thinness	Discrepancy between current and ideal figure: *t* = 3.67, *p* = 0.001. Mean discrepancy: female 0.56–1.02, male − 0.02-1.00 *M* (*SD*) = Female current 3.75 (0.9), ideal 3.19 (0.9). Male current 4.18 (1.1), ideal 4.2 (0.7)
Jordan	Madanat, Hawks, & Angeles (2011) [83]	*N* = 800, female	Cross sectional	9- figure silhouettes	Body dissatisfaction: 66%	not reported
Jordan	Mousa, Mashal, Al-Domi, & Jibril (2010) [84]	*N* = 326, female, age: 10–16	Cross sectional	EAT 26 BSQ |Western norms	Body dissatisfaction: 21.2%. Association between EAT and BSQ.	BSQ: *M* (*SD*) = 79.1 (34.5), 21.2% above cutoff χ2 (1, 326) = 104.8, *p* < 0.01
Jordan	Zawawi (2014) [85]	*N* = 170, female, age: 20–55, fitness center users	Cross sectional	BSQ	Body dissatisfaction: 31.01%	*M* (*SD*) = 3.19 (10.3)
Kuwait	Ebrahim, Alkazemi, Zafar, & Kubow (2019) [86]	*N* = 400, Male, university students	Cross sectional	Body Builder Image Grid	Body dissatisfaction: 69%, desire to lose body fat associated with disordered eating attitudes	OR = 1.898 [1.214–2.967], *p* = 0.005
Kuwait	Musaiger & Al- Mannai (2013) [26]	*N* = 228, female, university students, age 19–25	Cross sectional	Questions validated by Field et al., 2005, translated into Arabic	Body dissatisfaction: non-obese: 30%, obese: 81%. 21.6% of non-obese perceived themselves as overweight	not reported
Lebanon	Zeeni, Gharibeh, & Katsounari (2013) [23]	*N* = 400, female, university students in Cyprus (*n* = 200) and Lebanon (*n* = 200)	Cross sectional	Dutch eating behavior questionnaire	Association between restrained and emotional eating	*M* (*SD*): restrained = 29.20 (0.71), emotional eating = 37.76 (0.98), external eating = 33.33 (0.51), *p* < 0.05,
Qatar	Bener, Kamal, Tewfik, & Sabuncuoglu (2006) [35]	*N* = 800, male, age 14–19	Case control (dieting)	Adolescent dieting scale Self- reporting questionnaire	Extreme dieting: 10.1%	not reported
Qatar	Musaiger, Shahbeek, & Al-Mannai (2004) [15]	*N* = 535, male, age 20–67, primary health care center visitors	Cross sectional	9- figure silhouettes	Desire to be thin: 21.6%, low education 40%, mid-level education 45%, high education 53%. Desire to be thin was associated with age and education	Thin ideal, education: *p* = 0.0001, age > 40 years *p* = 0.0001
Saudi Arabia	Al- Subaie (2000) [87]	*N* = 1179, female, mean age = 16.1	Cross sectional	EDI 2 DT	Desire to be thin: 15.9%	*M* = 6.7, *SD* not reported
Saudi Arabia	Fallatah, Al-Hemairy, & Al-Ghamidi (2015) [66]	*N* = 425,female, age 15–18	Cross sectional	EAT 26	Prevalence of dieting not reported	Dieting: 9.38 (7.0)
UAE	Eapen, Mabrouk, & bin Othman (2006) [20]	*N* = 495, female, age 13–18	Cross sectional	EAT 40	Thin ideal: 66% preferred a slimmer body than their actual body. Desire to be thin associated with elevated EAT 40 scores	*p* < 0.0001
**Country**	**Authors (year)**	**Participants**	**Study design**	**Measures**	**Eating disorder-related variables**	**M (*SD*)/OR [95% C.I.]/ r**
UAE	O’Hara et al., (2016) [74]	*N* = 420, female, mean age = 23.12, university students	Cross sectional	EAT 26 Teasing frequency from Project eating attitudes and teens Weight and body related shame and guilt scale	Dieting associated with body dissatisfaction	*r* = 0.66, *p* < 0.001
UAE	Sawadi, Bener, & Darmaki (2000) [88]	*N* = 540, female, age 11–19	Cross sectional	Adolescent dieting scale	Dieting: 89.4% dieting, 9.1% extreme dieting	not reported
UAE	Schulte & Thomas (2013) [89]	*N* = 361, 284 female, 77 male, age 11–19, university students	Cross sectional	EAT 26	Body image dissatisfaction: 73%, female: 78%, male: 58%, body dissatisfaction associated with desire to be thin and elevated EAT score	Desire to be thin: χ2(2) = 27.083, *p* < 0.001, EAT: *t*(348), *p* < 0.001
UAE	Schulte (2016) [90]	*N* = 236, mean age = 19.78	Cross sectional	Body esteem scale, emotional eating scale, Weight and body related shame and guilt scale	Disturbed eating behavior and body dissatisfaction associated with binge eating	*M* (*SD*): body related shame = 8.00 (8.00), body related guilt = 11.50 (9.00). Associations with binge eating: disturbed eating behavior: *p* < 0.001, body dissatisfaction: *p* < 0.001
UAE	Thomas, Khan, & Abdulrahman (2010) [14]	*N* = 228, female, mean age = 19.8, university students	Cross sectional	EAT 26 FRS	Body dissatisfaction: 74.8%, association between body image dissatisfaction and disturbed eating behavior	*r* = 0.27, *p* = 0.01

Note: *BSQ* Body Shape Questionnaire, *EAT* Eating Attitude Test, *FRS* Figure Rating Scale, *EDI 2 DT* Eating Disorders Inventory 2 Drive for Thinness Scale

#### Disturbed eating behavior

Overall, the evidence suggested that disturbed eating behavior increases with age during adolescence and decreases during adulthood [20, 26, 29, 52, 53, 58, 67]. Females [26] and Saudis [67] appeared to be most at risk of disturbed eating behavior [14, 26, 90].

#### Dieting

The findings suggested that, as a whole, approximately 40% of the Arab population is on a diet [20, 35, 70, 88], and this is true for adults and adolescents of both genders [20, 35, 70]. Saudis were at the lowest risk of dieting [13].

#### Body dissatisfaction

Females were more vulnerable to body dissatisfaction than male Arabs [10, 82]. Higher BMI was associated with greater body dissatisfaction [64, 75, 83–85, 91].

#### Desire to be thin

Though findings were quite inconsistent, overall Arabs from the UAE had the highest desire to be thin [13–15], whereas Qataris appeared to be minimally affected by the thin ideal [15]. A risk factor of the desire to be thin was adulthood over adolescence [10, 13–15, 20, 87, 89].

### Eating disorder symptoms

Table 2 provides a summary of 11 studies that examined eating disorder symptoms in the Arab world. Binge eating and restrained eating behavior were each identified in seven cross sectional studies and compensatory behavior (*n* = 2) in a few studies. All ED symptoms were identified by self-report among adolescents: high school and/or university students.

**Table 2:** Summary of studies with focus on eating disorder symptoms in the Arab world

Country/ Population	Authors (year)	Participants	Study design	Measures	Eating disorder symptoms
Egypt	Dolan & Ford (1991) [11]	*N* = 218, mean age = 20, university students	Cross sectional	BSQ Restraint scale Binge scale	Binge eating: female 82%, male 76%
Jordan	Mousa, Mashal, Al-Domi, & Jibril (2010) [84]	*N* = 326, female, age: 10–16	Cross sectional	EAT 26 BSQ	Binge eating: 33%
Jordan	Mousa, Al-Domi, Mashal, & Jibril (2010) [77]	*N* = 432, female, age: 10–16	Cross sectional	EAT 26 BSQ Eating habits questionnaire	Binge eating: 16.9%, self-induced vomiting: 11%
Kuwait	El-Ghazali, Ibrahim, Kanari, & Ismail (2010) [92]	*N* = 320, 223 male, 97 female, mean age = 21.1	Cross sectional	Questionnaire for emotional eating (self- developed)	Emotional eating: female: 85.6%, male: 87.9%
Lebanon	Yahia, El- Ghazale, Achkar, & Rizk (2011) [93]	*N* = 252, female, students	Cross sectional	BSQ	Laxatives: 8%, diet pills: 4%
Lebanon	Zeeni, Gharibeh, & Katsounari (2013) [23]	N = 400, female, university students in Cyprus (n = 200) and Lebanon (n = 200)	Cross sectional	Dutch eating behavior questionnaire	Restrained eating Lebanese students: 30%
Jordan	Afifi- Soweid, Najem Kteily, & Shedia- Rizkallah (2001) [94]	*N* = 954, mean age = 18, university students	Cross sectional	Self-developed questionnaire	Binge eating: 4.9%, secretly overeating: 3.7%
Jordan	Mousa, Mashal, Al-Domi, & Jibril (2010) [84]	N = 326, female, age: 10–16	Cross sectional	EAT 26	Restrained: 40.5%
Oman	Al Adawi et al., (2002) [29]	*N* = 293, 106 teenagers, mean age = 15.12, 100 adults, mean age = 38.71, 87 Western teenagers resided in Oman, mean age = 15.10	Cross sectional	EAT Bulimic Investigatory Test	Binge eating: Omani teenagers 14%, Western teenagers 18% Restrained: 33% Omani teenagers, 9% Western teenagers
Palestinians residing in the Northern and Haifa district	Latzer, Azaiza, & Tzischinsky (2009) [70]	*N* = 928, female, age: 12–18, 81.2% Islamic, 11.2% Christian, 7.6% Druze	Cross sectional	EAT 26	Laxatives: 7%, self-induced vomiting: 8%
UAE	Schulte (2016) [90]	*N* = 236 Mean age = 19.78	Cross sectional	Body esteem scale Emotional eating scale Weight and body related shame and guilt scale	Body esteem scale: 24.4–35.6% Binge eating: moderate binge eating 16.9–24.9%, severe binge eating 6.4–13.2%, Emotional eating scale: emotional eating 23–24.5%

Note: *BSQ* Body Shape Questionnaire, *EAT* Eating Attitude Test

#### Binge eating

Kuwaiti and Egyptian Arabs appeared to be most at risk of binge eating. However, binge eating was only assessed among adolescents, and the findings with regard to gender were inconsistent [11, 29, 74, 77, 84, 90, 92].

#### Restrained eating behavior

Off the seven studies included in this synthesis that studied restrained eating behavior, all of them reported restrained eating behavior in about one-third of Arabic females. Arab adolescents who reported emotional eating and Arabs with obesity were particularly at risk of restrained eating behavior [20, 23, 29, 66, 67, 77, 87].

#### Compensatory behavior

Around 7.5% of female Arab adolescents reported usage of laxatives [70, 93].

### Eating disorders

Table 3 provides a summary of 27 studies that examined prevalence estimates of Arabs at high risk for EDs. ED-screening instruments were conducted among both males and females, and among adolescents and adults. Arabs at high risk for anorexia nervosa (AN) and bulimia nervosa (BN) were identified in different countries by seven cross-sectional studies. Only one study reported Arabs at high risk for binge eating disorder (BED) and other specified feeding or eating disorders (OSFED) [90]. While most ED assessment tools were self-report measures, in one cross sectional study participants were interviewed using a self-developed interview [39]. Another study used the Structured Clinical Interview for DSM-IV (SCID) [76]. In one case control study among health care clinic attendees, EDs were not assessed [69]. Scores above a clinical cut-off on ED- screening instruments were reported without classification specification in 18 studies (Table 3).

**Table 3:** Summary of studies reporting the prevalence of Arabs at high risk for eating disorders

Country/ Population	Authors (year)	Participants	Study design	Measures| Norms	Elevated scores at eating disorder screeners ***M (SD)***
Algeria, Jordan, Kuwait, Libya, Palestinians residing in al-Khalil, Syria, UAE	Musaiger et al., (2013) [26]	*N =* 4698, 2240 male, 2458 female, age 15–18	Cross sectional	EAT 26| Saudi norms	Above clinical cut off: male:13.8–47.3%, female: 16.2–42.7% Algeria: 15.2%, male 13.8, female 16.2% Jordan: 31.6%, male 20.1%, female 42.7% Kuwait: 44.7%, male 47.3%, female 42.8% Libya: 26.7%, male 19.3%, female 32.6% Palestinians residing in al-Khalil: 31.7%, male 23.2%, female 38.9% Syria: 22.9%, male 14.6%, female 32% UAE: 33.5%, male 29.8% female 37.4% *M (SD)* not reported
Egypt	Eladawi et al., (2018) [28]	*N =* 400, 112 male, 288 female, weight control center visitors	Cross sectional	EAT 40| Western norms	65% above clinical cut off *M (SD)* = 45.2 (10.2) (age 25–66)
Egypt	Nasser (1994) [39]	*N =* 35, female, age 15–16, secondary school students	Cross sectional	EAT 40| Western norms Eating interview	EAT 40: 11.4% above clinical cut off *M (SD)* total sample not reported Eating interview: 1.2% BN, 3.4% subclinical BN
Iran	Abdollahi & Mann (2001) [64]	*N =* 114, female, university students, Iranian nationals, 45 resided in LA, 59 resided in Teheran	Cross sectional	EDE-Q FRS	% above clinical cutoff not reported
Iran	Raouf et al., (2015) [76]	*N =* 1990, 951 male, 1039 female, age 13–18, mean age = 15.8	Cross sectional	EAT 26| Irani norms SCID	EAT 26: 24.2% above clinical cut off *M (SD)* not reported SCID: 0.25% diagnosed with ED, 0.7% AN, 0.9% BN, 1.0% OSFED
Jordan	Madanat et al., (2007) [80]	*N =* 800, female, mean age = 33.5	Cross sectional	EAT 26| Saudi norms Motivation for eating scale Restraint scale Sociocultural attitudes towards appearance scale Body esteem scale 9- figure silhouettes	EAT 26: 54.8% above clinical cut off *M (SD)* = 18.98 (10.76)
Jordan	Mousa, Mashal, Al-Domi, & Jibril (2010) [84]	*N =* 326, female, age: 10–16	Cross sectional	EAT 26| Western norms BSQ| Kuwaiti norms	EAT 26: 40.5% above clinical cut off *M (SD)* = 16.6 (10.7)
Jordan	Mousa, Al-Domi, et al., (2010) [77]	*N =* 432, female, age: 10–16	Cross sectional	Eating habits questionnaire	OSFED: 31%, BED: 1.8%, BN: 0.6%, AN: 0% *M (SD)* not reported
Kuwait	Alkhadari et al., (2016) [69]	*N =* 1046, 429 male, 617 female, mean age = 37.6, health care clinic attendees	Case control (health care clinic attendees)	Patient health questionnaires GAD-7	Eating disorders were not assessed
Kuwait	Ebrahim, Alkazemi, Zafar, & Kubow (2019) [86]	*N =* 400, male, university students	Cross sectional	EAT 26| Saudi norms	46.2% above clinical cut off *M (SD)* = 20.4 (14.1)
Lebanon	Aoun et al., (2015) [54]	*N =* 123, female, age 15–55, primary health care center visitors	Validation SCOFF	SCOFF	28% above clinical cut off *M (SD)* not reported
Oman	Kayano et al., (2008), Viernes et al., (2007) [52, 58]	*N =* 248, 135 Omani, 113 Westerners resided in Oman, age 13–18	Cross sectional	EAT 26| Saudi norms EDI 2 DT| Saudi norms	% above cut-off not reported EAT: Omani: *M (SD)* = 8.48 (1.64), European expatriates: *M (SD)* = 5.98 (1.83) EDI2 DT: Omani: *M (SD)* = 4.12 (0.60), European expatriates: *M (SD)* = 10.14 (0.64).
**Country**	**Authors (year)**	**Participants**	**Study design**	**Measures| Norms**	**Elevated scores at eating disorder screeners** ***M (SD)***
Oman	Al Adawi et al., (2002) [29]	*N =* 293, 106 teenagers, mean age = 15.12, 100 adults, mean age = 38.71; 87 Western teenagers resided in Oman, mean age = 15.10	Cross sectional	EAT 26| Saudi norms Bulimic Investigatory Test	EAT: 33% Omani teenagers, 9% Western teenagers above clinical cut off *M (SD)* not reported Bulimic Investigatory Test: 12.3% Omani teenagers, 2% Omani adults, 18.4% Western teenagers
Palestinians residing in the Northern and Haifa district	Latzer et al., (2009) [70]	*N =* 1141, female, age: 12–18, 81.2% Islamic, 11.2% Christian, 7.6% Druze	Cross sectional	EAT 26| Saudi norms	25% above clinical cut off *M (SD)* age 12–13 = 16.5 (11.9), age 14–15 = 15.0 (10.5), age 16–18 = 15.2 (9.7)
Palestinians residing in Nablus	Saleh et al., (2018) [36]	*N =* 2001, female university students	Cross sectional	EAT 26| Saudi norms SCOFF	EAT: 28.6% above clinical cut off *M (SD)* = 15.27 (10.38) SCOFF: 38.2% above clinical cut off *M (SD)* = 1.25 (1.032)
Qatar and Lebanon	Kronfol et al., (2018) [55]	*N =* 1841, 167 Lebanon, 785 Qatar, 889 USA university students	Cross sectional	SCOFF	Arab students: 20.4% above clinical cut off American students: 6.8% above clinical cut off *M (SD)* not reported
Saudi Arabia	Al- Subaie (2000) [87]	*N =* 1179, female, mean age = 16.1	Cross sectional	EDI 2 DT	15.9% above clinical cut off M 6.7 SD not reported
Saudi Arabia	Bano et al., (2013) [67]	*N =* 100, female, age 18–25	Cross sectional	EAT 26| Western norms	Female: 24% above clinical cut off, male: 2% above clinical cut off *M (SD)* female = 16.89 (10.52), *M (SD)* male = 9.88 (13.26)
Saudi Arabia	Fallatah et al., (2015) [66]	*N =* 425, female, age 15–18	Cross sectional	EAT 26| Saudi norms	32.9% above clinical cut off *M (SD)* = 17.98 (9.29)
UAE	Eapen et al., (2006) [20]	*N =* 495, female, age 13–18	Cross sectional	EAT 40| Western norms	23.4% above clinical cut off *M (SD)* = 15.19 (1.94)
UAE	Musaiger, Al-Mannai, & Al-Lalla (2014) [79]	*N =* 731, male, age 15–18, resided in 5 different Emirates	Cross sectional	EAT 26| Saudi norms	% above clinical cut off: Dubai 49.1%, Ajman 33.1%, Al Fujairah 48.0%, Ras al Khaima 34.8%, Um al Quain 39.7% *M (SD)* not reported
UAE	O’Hara et al., (2016) [74]	*N =* 420, female, mean age = 23.12, university students	Cross sectional	EAT 26| Western norms Teasing frequency from Project eating attitudes and teens Weight and body related shame and guilt scale	EAT 26: 30% above clinical cut off *M (SD)* = 15.57 (9.03)
UAE	Schulte & Thomas (2013) [89]	*N =* 361, 77 male, 284 female, age 11–19	Cross sectional	EAT 26| Western norms	20% above clinical cut off *M (SD)* female = 12.88 (8.91), *M (SD)* male = 11.21 (9.81)
UAE	Thomas et al., (2010) [14]	*N =* 228, female, mean age = 19.8, university students	Cross sectional	EAT 26| Western norms	24.6% above clinical cut off *M (SD)* = 13.31 (10.21)
UAE	Thomas, O’Hara, et al., (2018) [53]	*N =* 1069, female, university students	Cross sectional	EAT 26| Western norms	29.0% above clinical cut off *M (SD)* = 15.80 (9.39)
UAE	Thomas, O’Hara, et al., (2018) [18]	*N =* 209, female, university students	Cross sectional	EAT 26| Western norms	30.3% above clinical cut off *M (SD)* = 14.17 (9.40)

Note: *BSQ* Body Shape Questionnaire, *EAT* Eating Attitude Test, *EDE-Q* Eating Disorder Examination Questionnaire, *FRS* Figure Rating Scale, *EDI 2 DT* Eating Disorders Inventory 2 Drive for Thinness Scale, *SCID* Structured Clinical Interview for DSM IV, *SCOFF* Sick, Control, One, Stone, Fat, Food

#### Arabs at high risk for ED

In general, 13–55% was at high risk for EDs. On ED-screening instruments, females (11.4–54.8%) displayed more to be at high risk for EDs than males (2–47.3%) [16, 18, 20, 26, 28, 29, 36, 39, 40, 52–55, 58, 64, 66, 67, 69, 70, 74, 76, 77, 79, 80, 84, 87, 89, 91]. Arabs from the UAE appeared to be more at risk for the development of EDs than Arabs from other countries [12, 29, 76, 77].

### Correlates

Table 4 presents a summary of 32 studies on correlates of a high risk for EDs, ED symptoms and ED-related variables. In Western countries, increased affluence and obesity appear to be the major correlates [44]. These factors may also play a role in Arab communities [76], as the Arab world experiences Western and media influences (social media, TV, western advertising and magazines) [84], which may be correlates of the development of EDs [23]. Increased affluence was identified in five cross sectional studies, Western influences in 11 cross sectional studies, media use in five cross sectional studies, and obesity in nine cross-sectional studies. Most were identified by self-report. However, one study investigated the association between media use and EDs in several countries with a self-developed interview [27], and another study examined EDs with the SCID [76].

**Table 4:** Summary of studies reporting correlates of eating disorders and eating disorder-related variables in the Arab world

Country/Population	Authors (year)	Participants	Study design	Measures	Risk factors	***M (SD)/*** OR [95% CI]/ ***r***
Algeria, Jordan, Kuwait, Libya, Palestinians residing in al-Khalil, Syria, UAE	Musaiger et al., (2013) [26]	*N =* 4698, 2240 male, 2458 female, age 15–18	Cross sectional	EAT 26	Obesity: disturbed eating behavior 2–3 times as high in obese than in non-obese males and females	*p* < 0.000
Bahrain, Egypt, Jordan, Oman, Syria	Musaiger (2014) [27]	*N =* 1134, female, university students, age 17–32	Cross sectional	Interview (self- developed)	Media use: exposure to magazines associated with dieting to lose weight in Bahrain, exposure to TV associated with desire to be thin in Egypt, Oman and Jordan, exposure to TV associated with dieting to lose weight in Egypt and Bahrain	Associations: magazines and dieting: Bahrain: OR = 2.29 [0.95–5.68], *p* < 0.044, Egypt OR = 6.29 [2.21–17.39], *p* < 0.001, Jordan OR = 5.29 [1.78–16.83], *p* < 0.001. TV and desire to be thin: Egypt OR = 2.05 [1.07–3.94], *p* < 0.019, Oman *p* < 0.019, OR = 2.41[1.09–5.48]. TV and dieting: Bahrain OR = 1.98 [1.00–3.94], *p* < 0.035, Egypt OR = 2.21 [1.01–4.92], *p* < 0.032
Egypt	Eladawi et al., (2018) [28]	*N =* 400, 112 male, 288 female, weight control center visitors	Case control	EAT 40	Increased affluence, female, rural residents, overweight, obesity associated with elevated EAT scores	Rural residents: OR = 1.75 [0.95–3.22], *p* = 0.044, affluence: OR = 3.17 [0.74–13.63], *p* = 0.023, weight: *p* = 0.006; overweight OR = 2.75 [1.42–5.33], obesity OR = 1.46 [0.82–2.59]
Iran	Abdollahi & Mann (2001) [64]	*N =* 114, female, university students, Iranian nationals, 45 resided in LA, 59 resided in Teheran	Cross sectional	EDE-Q	Western influences: difference between actual and desired BMI larger in LA sample than in Irani sample	*p* < 0.05
Iran	Raouf et al., (2015) [76]	*N =* 1990, 951 male, 1039 female, age 13–18, mean age = 15.8	Cross sectional	EAT 26 SCID	BMI, age, increased affluence, female gender associated with elevated scores	Female: EAT OR = 2.52 [0.42–0.65], *p* < 0.001, AN *p* < 0.001, BN *p* < 0.05, OSFED *p* < 0.001, age: OR = 1.09 [0.99–1.17], *p* = 0.036, BMI: OR = 0.93 [0.90–0.96], *p* < 0.001, increased affluence: OR = 1.17 [1.01–1.35], *p* = 0.029
Jordan	Madanat et al., (2007) [80]	*N =* 800, female, mean age = 33.5	Cross sectional	EAT 26 Motivation for eating scale Restraint scale Sociocultural attitudes towards appearance scale Body esteem scale 9- figure silhouettes	Weight status: 53.8% overweight/ obese. Obesity associated with desire to lose weight, restrained eating, emotional eating, elevated EAT scores. Western advertising and media use associated with desire to lose weight, restrained eating, emotional eating, disturbed eating behavior. Increased affluence associated with elevated EAT scores	Obesity: *p* < 0.01 Increased affluence: *p* < 0.01. Associations Western advertising and media not reported.
Jordan	Madanat, Hawks, & Angeles (2011) [83]	*N* = 800, female	Cross sectional	9- figure silhouettes	BMI: 53.8% overweight/ obese, 66% body dissatisfaction, association between BMI and desire to lose weight	*r* = 0.858, *p* < 0.0001
Jordan	Mousa, Mashal, Al-Domi, & Jibril (2010) [84]	*N =* 326, female, age: 10–16	Cross sectional	EAT 26 BSQ	Media use associated with body dissatisfaction. BMI associated with body dissatisfaction. Residing in a Western country is protective factor for body dissatisfaction.	Obesity: OR = 2.8 [2.1–3.8], *p* < 0.01 Media: OR = 1.2 [1.1–1.4], *p* < 0.01, reside in Western country: [RR: 0.34 (0.12–1.1), *p* = 0.046].
Jordan	Zawawi (2014) [85]	*N =* 170, female, age: 20–55, fitness center users	Cross sectional	BSQ	BMI: positive association between BMI and body dissatisfaction	*r* = 0.729, *r*^*2*^ = 0.53, *F*(1, 175) = 198.6, *p* < 0.01
**Country**	**Authors (year)**	**Participants**	**Study design**	**Measures**	**Risk factors**	***M (SD)/*** **OR [95% CI]/** ***r***
Kuwait	Ebrahim, Alkazemi, Zafar, & Kubow (2019) [86]	*N* = 400, Male, university students	Cross sectional	EAT 26	Obesity associated with disordered eating and dieting.	Disordered eating: OR = 2.06 [1.17, 3.60], *p* = 0.011, Dieting: OR = 2.063[1.01, 4.21], *p* = 0.043)
Kuwait	Musaiger & Al- Mannai (2013) [75]	*N =* 228, female, university students, age 19–25	Cross sectional	Questions validated by Field et al., 2005, translated into Arabic	Use of internet and reading magazines associated with dieting to lose weight, media influence 2–3 times higher in obese than in non-obese females, watching TV not associated with body shape concern.	Dieting: OR = 3.11 [1.5–6.47], Media influence: OR = 2.14 [0.93–5.09], internet: *p* = 0.000, magazines: *p* = 0.011, media influence in obese: *p* = 0.000
Lebanon and Cyprus	Zeeni, Gharibeh, & Katsounari (2013) [23]	*N* = 400, female, university students in Cyprus (*n* = 200) and Lebanon (*n* = 200)	Cross sectional	Dutch eating behavior questionnaire Perceived sociocultural influences on body image and body change questionnaire	Lebanese students greater association between body image dissatisfaction and media use. Greater emotional eating and sociocultural influences in eating behavior, greater influence of media to become slimmer, eat less and exercise to lose weight (*p* < 0.05). Association between BMI and restraint and emotional eating in Lebanon and in Cyprus. No differences in restraint eating	Associations Lebanon: BMI and restrained *r* = 0.3, *p* < 0.001, BMI and emotional eating *r* = 0.2, *p* = 0.01, media to become slimmer *t*(371.66) = 5.02, *p* < 0.001, *r* = 0.25), eat less to lose weight *t*(383.31) = 3.02, *p* < 0.001, *r* = 0.15, exercise more to lose weight *t*(380.90) = 3.53, *p* < 0.001, *r* = 0.18
Oman	Al Adawi et al., (2002) [29]	*N =* 293, 106 teenagers, mean age = 15.12, 100 adults, mean age = 38.71; 87 Western teenagers resided in Oman, mean age = 15.10	Cross sectional	EAT Bulimic Investigatory Test	Westernization: significant difference in BMI between Omani and Western teenagers, Omani teenagers significantly more susceptible for AN and BN than Western teenagers	BMI: *p* = 0.000
Oman	Kayano et al., (2008) [58]	*N =* 248, 135 Omani, 113 Westerners resided in Oman, age 13–18	Cross sectional	EAT 26 EDI 2 DT	Weight status: 13% obese, 27% underweight. BMI associated with desire to be thin, negative association between EDI and EAT 26 scores. Average score on EAT 26 higher in the Omani (9.2) than in the Western (5.59) group. EDI score 3 times higher in Western than in Omani group.	BMI: *r* = 0.03, *p* < 0.05, Omani higher EAT scores: *F*(2,240) = 10.95, *p* < 0.001. Europeans higher EDI 2 DT scores: *F*(2,240) = 71.72, *p* < 0.001
Oman	Viernes et al., (2007) [52]	*N* = 248, 135 Omani, 113 Westerners resided in Oman, age 13–18	Cross sectional	EAT 26 EDI 2 DT	BMI associated with desire to be thin and guilt after eating sweets. Terrified to become fat: European expats: 81%, Omani’s: 66%. higher fear of fatness. Somatic symptom presentation among Omani’s.	Desire to be thin: Omani OR = 1.60 [0.92 2.79], *p* = 0.09, European expats: OR = 8.17 [4.63 14.41], *p* = 0.00, guilt after eating sweets: Omani OR = 0.05 [0.01 0.36], *p* = 0.00. Terrified to become fat: *F* = 235.9, *p* < 0.001. Somatic symptom presentation: Kendall’s tau = 0.352, *p* < 0.001
Palestinians residing in the Northern and Haifa district	Latzer et al., (2009) [70]	*N =* 1141, female, age: 12–18, 81.2% Islamic, 11.2% Christian, 7.6% Druze	Cross sectional	EAT 26	Westernization: Druze subgroup higher scores on EAT	*F* [2] = 2.9, *p* < 0.05
Palestinians residing in Nablus	Saleh et al., (2018) [36]	*N =* 2001, female university students	Cross sectional	EAT 26 SCOFF	BMI: association between BMI and EAT score. Age: negative association between age and EAT score	BMI: *r* = 0.173, *p* < 0.011 age: *r* = − 0.058, *p* = .008
Qatar	Bener & Kamal (2006) [35]	*N* = 566, female, age 14–19	Cross sectional	Adolescent dieting scale	BMI associated with dieting	*p* = 0.045
Qatar	Bener, Kamal, Tewfik, & Sabuncuoglu (2006) [35]	*N* = 800, male, age 14–19	Case control (dieting)	Adolescent dieting scale Self- reporting questionnaire	Obesity: 34% of dieters was overweight, TV was diet source (61.7%)	*p* = 0.014
**Country**	**Authors (year)**	**Participants**	**Study design**	**Measures**	**Risk factors**	***M (SD)/*** **OR [95% CI]/** ***r***
Qatar	Musaiger, Shahbeek, & Al-Mannai (2004) [15]	*N* = 535, male, age 20–67, primary health care center visitors	Cross sectional	9- figure silhouettes	Age and education associated with desire to be thin	Association with desire to be thin: education: *p* = 0.0001, age > 40 years *p* = 0.0001
Qatar and Lebanon	Kronfol et al., (2018) [55]	*N =* 1841, 167 Lebanon, 785 Qatar, 889 USA university students	Cross sectional	SCOFF	Risk factors: female gender, financial difficulties	*p* < 0.001
Saudi Arabia	Al- Subaie (2000) [87]	*N =* 1179, female, mean age = 16.1	Cross sectional	EDI 2 DT	BMI, speaking a Western language and lived in a Western country and SES associated with dieting behavior and drive for thinness	BMI: χ2(3) = 97.59, *p* = 0.0001, western language χ2(1) = 8.9, *p* = 0.002, lived in western country χ2(1) = 10.3, *p* = 0.001, SES χ2(4) = 12.32, *p* = 0.015
Saudi Arabia	Fallatah et al., (2015) [66]	*N =* 425, female, age 15–18	Cross sectional	EAT 26	Association between BMI and disturbed eating behavior	*t* = 3.095, *p* < 0.0001, df not reported
Saudi Arabia	Khalaf, Westergren, Berggren, Ekblom, & Al-Hazzaa (2015) [95]	*N* = 663, female, mean age = 20.4, university students	Cross sectional	Self-developed questionnaire	Weight status: 19.2% underweight, 56.9% normal weight, 23.8% overweight/ obesity, BMI was associated with increased affluence	*p* = 0.032
Saudi Arabia	Rasheed (1998) [68]	*N* = 144, female, 74 Obese, 70 non- obese, age 15–55	Case control study (obesity)	Adapted eating and exercise behavior questionnaire	Increased affluence: higher affluence leads to slimmer ideal body (81%) and overestimation of own body weight (29%), illiteracy more common in obese group (21.9%).	*p* < 0.05
UAE	Eapen et al., (2006) [20]	*N =* 495, female, age 13–18	Cross sectional	EAT 40	BMI, age, Western TV associated with elevated EAT scores.	*p* < 0.0001
UAE	O’Hara et al., (2016) [74]	*N =* 420, female, mean age = 23.12, university students	Cross sectional	EAT 26 Teasing frequency from Project eating attitudes and teens Weight and body related shame and guilt scale	Internalized weight stigma and teased with weight associated with elevated EAT scores	Internalized weight stigma: *r* = 0.43, *p* < 0.001, teased with weight: *r* = 0.19, *p* = 0.008
UAE	Schulte & Thomas (2013) [89]	*N =* 361, 77 male, 284 female, age 11–19, university students	Cross sectional	EAT 26	Weight status: overweight: 18.6%, obesity 9.2% BMI associated with EAT scores in females	BMI: *r* = 0.184, *p* = 0.005
UAE	Schulte (2016) [90]	*N* = 236, mean age = 19.78	Cross sectional	Body esteem scale PSS Emotional eating scale, Weight and body related shame and guilt scale	After correcting for BMI association between perceived stress and binge eating	*p* = 0.043
UAE	Thomas, Quadflieg, & O’Hara (2016) [40]	*N =* 94, female, university students	Cross sectional	EAT 26	Implicit out group preference associated with elevated EAT scores	*t* [91] = 2.83, *p* < 0.001
UAE	Thomas, O’Hara, et al., (2018) [53]	*N =* 1069, female, university students	Cross sectional	EAT 26	Religiosity: small effect size for religiosity in the group that scored above clinical cut off on the EAT	*U* = 91,660, *p* < 0.001, *r* = − 0.12
UAE	Thomas, O’Hara, et al., (2018) [18]	*N =* 209, female, university students	Cross sectional	EAT 26	Westernization: small effect size for out group positivity and higher Western acculturation in the group that scored above clinical cut off on the EAT	Out group positivity: *t*(206) = 2.49, *p* = 0.013, *d* = 0.36. Western acculturation: *t*(206) = 3.13, *p* = 0.002, *d* = 0.46

Note: *BSQ* Body Shape Questionnaire, *EAT* Eating Attitude Test, *EDE-Q* Eating Disorder Examination Questionnaire, *FRS* Figure Rating Scale, *EDI 2 DT* Eating Disorders Inventory 2 Drive for Thinness Scale, *SCID* Structured Clinical Interview for DSM IV, *SCOFF* Sick, Control, One, Stone, Fat, Food

#### Increased affluence

Increased affluence was associated with ED-related variables [16, 90, 96], especially among Saudis [24, 62, 69, 90].

#### Western influences

Exposure to Western influences was associated with ED-related variables, especially the desire to be thin [13, 22, 37, 70]. Examples of such Western influences were media [14, 31], traveling abroad [35], living abroad [90, 92, 97], and contact with expatriates [13, 18, 35, 37]. Arabs with greater assimilation with the Western culture were potentially at more at risk for EDs [22]. Arabs in Gulf countries (Oman, Saudi Arabia, UAE) were particularly vulnerable to Western influences. However, besides Western influences vulnerability for EDs might also be associated with industrialization, as the Gulf also has the highest level of industrialization within the Arab world [13, 37].

#### Media use

Usage of social media was associated with ED symptoms [98, 99], greater body dissatisfaction, the desire to be thin [100–102], and disturbed eating behavior [94, 103]. Media use was a particular risk factor among adolescents [18, 23, 69, 74, 83].

#### Obesity

A higher BMI was positively associated with ED-related variables [74, 104–106], ED symptoms [24, 62] and high risk for EDs [22]. This is of concern because the Arab world has one of the highest rates of obesity in the world [107], in Saudi Arabia in particular [108], followed by Bahrain, Egypt, Jordan, Syria and Oman [91].

### Cultural differences in eating disorders

Some studies suggest that, rather than causing a rise in EDs, Western influences are associated with a shift in symptomatology. The four cross-sectional studies summarized in Table 4 examined cultural differences in EDs.

#### Cultural differences

Interestingly, predominant identification with a Middle Eastern culture was associated with a risk for AN [29, 64], while predominant identification with a Western culture was associated with a risk for BN [64], for instance among European expatriates resident in Oman, among Omani Arabs [29] and among Iranians [64]. Omani Arabs tended to express their ED symptoms somatically (e.g. bloated stomach, nausea, reduced, fluttering feeling in the stomach, throat discomfort, etc.), while Western populations were more likely to express them psychiatrically [52, 58].

## Discussion

The aim of this study was to provide estimates regarding the point-prevalence of Arabs at high risk for EDs, with ED symptoms and ED-related variables. An additional aim was to provide a comprehensive synthesis of relevant studies of correlates and ED-related variables. Although EDs occur both in Western [60] and in Arab societies [76], there are no official statistics available for in the Arab world [96] and prevalence has rarely been studied [90]. The prevalence of full syndrome EDs have not been reported in the Arab world, except for one case study [97]. Perhaps this is because EDs are not viewed as common disorders in the Arab world [69]. This synthesis found that in the Arab world, 13–55% is at high risk for EDs, the prevalence was higher among females than in males [16, 18, 20, 26, 28, 29, 36, 39, 40, 52–55, 58, 64, 66, 67, 69, 70, 74, 76, 77, 79, 80, 84, 87, 89, 91], and the prevalence appeared to increase during adolescence [20, 76, 98, 99]. There were also some indications symptomatology was culturally reactive [12, 29, 52, 58, 64, 87].

EDs are perceived as diseases of globalization [6]. The rapid sociocultural changes in the Arab world [20] since oil boom in the 1970s and the consequent increased affluence have contributed to elevated levels of non-communicable diseases including the development of ED-related variables, ED symptoms and EDs [18, 38–40]. In the included studies sociocultural changes were associated with changes in types of food available (higher in salt, fat and sugar) and therefore increased prevalence of obesity [34, 100–102]. Obesity was associated with dieting behavior, compensatory behavior, body dissatisfaction and binge eating behavior [13, 20, 23, 26, 70, 76, 85, 103]. Another implication of the sociocultural changes was the shift from admiration of a curvy body [23] to that of a thin body [10, 30, 80, 94], and the desire to be thin was associated with compensatory behavior, binge eating behavior, dieting behavior, and body dissatisfaction [13, 20, 23, 26, 70, 76, 85, 103]. The thin ideal was especially prevalent among adults [10, 13–15, 20, 87, 89], higher educated, increased affluence Arabs [38, 104], and Arabs who reported Western influences [18, 30]. Interestingly, societies dealing with faster industrialization appeared more vulnerable to Western influences [18, 40] and displayed greater risk for EDs [12, 29, 76, 77]. However, Qatari females appeared minimally affected by the thin ideal [15]. There were indications that culture was associated with symptomatology [29, 52, 58, 64], and the somatic symptom presentation [52, 58] was in line with other countries dealing with rapid sociocultural changes [59, 105, 106].

In conclusion, female Arabs were most at risk for EDs and ED symptoms [20, 26, 29, 52, 53, 58, 67]. The high rates of obesity [34, 100–102] and the desire to be thin were associated with other ED-related variables, ED symptoms and high risk for EDs [13, 20, 23, 26, 70, 76, 85, 103]. Increased affluence and media use were correlates of the desire to be thin.

### Clinical impact

Development of this synthesis of EDs in the Arab world has important clinical implications, as most studies examining ED prevalence used Western assessment tools [90]. Such utilization of potentially insensitive assessment tools and potentially inappropriate norms may lead to underestimation of symptom severity [107], elevated rates of undiagnosed EDs, and to a lack of knowledge and (public) awareness about EDs and its correlates [108]. This in turn hampers timely and proper treatment of EDs [69]. In the Arab world, the situation is further complicated by the lack of specialized therapists and treatment facilities [97].

Another problematic aspect of the lack of popular knowledge about EDs is stigmatization, leading to delayed help seeking [108]. Since it is culturally unacceptable to discuss personal matters with non-family members, only 0.3% of Arabs with an ED seek help, compared to 20% of Westerners who do so [70]. More knowledge about EDs might decrease the current preference for self- or family treatment, and therefore stigma associated with psychotherapy [70]. In order to increase knowledge psycho-education can be offered at high-schools, including a parental program. Once college and high-school social workers are better educated in recognizing EDs they can refer their students to mental health care clinics. In addition, overweight Arabs tend to seek bariatric surgery, while currently they are in general not screened for EDs. Screening for EDs and refer Arabs at high risk for EDs might reduce delayed help seeking. Last, to decrease stigmatization, it might be beneficial to let influencers share their personal story of recovery of an ED, since Arabs are extensive social media users.

Two studies in Oman reported that, as in several Asian studies [59, 105, 106], EDs were explained somatically rather than psychiatrically [52, 58]. This may lead to a failure to accurately recognize behaviors as ED symptoms, and so to delayed help-seeking and greater symptom severity [109, 110]. In addition, in Saudi Arabia people with EDs tended to seek help only after experiencing somatic complaints, such as kidney failure or diabetes mellitus.

Although EDs have been studied in the Arab world, there are methodological shortcomings to many of the studies currently available. Arabs who display an increased risk for EDs, are likely to benefit from preventative programs, as selective prevention programs display larger effects than universal prevention programs [49, 111–113]. The risk groups in the Arab world are individuals with elevated levels of body dissatisfaction, desire to be thin, and self-reported dieting [13, 20, 23, 26, 70, 76, 85, 103]. In addition, the more high affluent societies and Arabs with extensive (social) media use, and more prone to Western influences are also at risk [38, 104]. Counteracting the elevated prevalence of obesity in order to prevent associated health risks [80], accompanied by psycho-education in order to prevent maladaptive weight-loss strategies, may be beneficial.

### Future directions

Most assessment tools included in this synthesis have been devised for use in Western populations. Due to the potential bias, it is recommended to use ED-screening instruments adapted for use in the Arab world. Future research might therefore consider the development and validation of cultural sensitive assessment tools [66]. One relevant adaptation of self- report measures is that fasting may be motivated by religion [77, 96] and should only be considered pathological if motivated by weight and shape concern. Other relevant adaptations in some Arab countries might relate to the strict gender separations and related cultural norms and behaviors, e.g., a proper attention to distinguishing between situations where females do and don’t cover themselves [77].

Another potential bias of the studies included in this synthesis is the usage of Western norms. In general, scores on ED-screening instruments are significantly lower in Western populations than in Arab populations [28, 80, 114, 115], usage of Western norms might therefore reflect bias. While some assessment tools have been validated in other Arab countries, they potentially may not be sensitive to nuances across various Arab communities. Efforts to assess EDs in the Arab world have therefore potentially been limited due to the absence of psychometric validation of assessment tools, especially in clinical samples. In order to understand the culture, validation studies should be supplemented with interview data. A culturally sensitive assessment tool will help minimize inconsistencies found among studies in the Arab world.

To elucidate correlates and ED-related variables, researchers need to examine levels of acculturation, internalization of the thin ideal, increased affluence and media exposure, as they may mediate body image dissatisfaction and disturbed eating behavior. As most studies included in this synthesis were conducted in countries dealing with rapid sociocultural changes, additional studies in the Arab world with limited exposure to Western influences and limited industrialization are necessary [116, 117].

Recently, studies are beginning to address the possible somatic symptom presentation of EDs in the Arab world [52, 58]. We recommend that, during diagnostic interviews, clinicians pay attention to the possibility of this understudied phenomena. In addition, it is worth further investigating the somatic attribution of EDs in the Arab world. Confirmation of this theory will facilitate recognition of EDs in the Arab world.

## Supplementary information

**Additional file 1.**

## Data Availability

Not applicable.

## Abbreviations

AN: Anorexia Nervosa
BED: Binge Eating Disorder
BES: Binge Eating Scale
BMI: Body Mass Index
BN: Bulimia Nervosa
BSQ: Body Shape Questionnaire
EAT: Eating Attitudes Test
ED: Eating Disorders
EDE-Q: Eating Disorder Examination Questionnaire
EDI 2 DT: Eating Disorders Inventory 2 Drive for Thinness Scale
FRS: Figure Rating Scale
GAD-7: General Anxiety Disorder questionnaire
OCI-R: Obsessive Compulsive Inventory
OSFED: Other Specified Feeding or Eating Disorder
PRISMA: Preferred Reporting Items for Systematic reviews and Meta-Analyses
PSS: Perceived Stress Scale
SCID: Structured Clinical Interview for DSM IV
SCOFF: Sick, Control, One, Stone, Fat, Food
UAE: United Arab Emirates
