# Supplementary material for: Eating disorders in the Arab world: a literature review
Source: J Eat Disord. 2020 Nov 6;8:59. doi: 10.1186/s40337-020-00336-x (PMC7646071; doi:10.1186/s40337-020-00336-x)
Supplement: Supplementary file 1 — Additional file 1. [file 40337_2020_336_MOESM1_ESM.docx]

**NEWCASTLE - OTTAWA QUALITY ASSESSMENT SCALE (adapted for cross sectional studies)** (Modesti et al., 2016)

**Selection: (Maximum 5 stars)**

1) Representativeness of the sample:

a) Truly representative of the average in the target population. * (all subjects or random sampling)

b) Somewhat representative of the average in the target population. * (nonrandom sampling)

c) Selected group of users.

d) No description of the sampling strategy.

2) Sample size:

a) Justified and satisfactory. *

b) Not justified.

3) Non-respondents:

a) Comparability between respondents and non-respondents characteristics is established, and the response rate is satisfactory. *

b) The response rate is unsatisfactory, or the comparability between respondents and non-respondents is unsatisfactory.

c) No description of the response rate or the characteristics of the responders and the non-responders.

4) Ascertainment of the exposure (risk factor):

a) Validated measurement tool. **

b) Non-validated measurement tool, but the tool is available or described.*

c) No description of the measurement tool.

**Comparability: (Maximum 2 stars)**

1) The subjects in different outcome groups are comparable, based on the study design or analysis. Confounding factors are controlled.

a) The study controls for the most important factor (select one). *

b) The study control for any additional factor. *

**Outcome: (Maximum 3 stars)**

1) Assessment of the outcome:

a) Independent blind assessment. **

b) Record linkage. **

c) Self-report. *

d) No description.

2) Statistical test:

a) The statistical test used to analyze the data is clearly described and appropriate, and the measurement of the association is presented, including confidence intervals and the probability level (p value). *

b) The statistical test is not appropriate, not described or incomplete.

This scale has been adapted from the Newcastle-Ottawa Quality Assessment Scale for cohort studies to perform a quality assessment of cross-sectional studies for the systematic review, “Eating Disorders in the Arab world: a literature review”.

Reference

Modesti, P. A., Reboldi, G., Cappuccio, F. P., Agyemang, C., Remuzzi, G., Rapi, S., . . . Parati, G. (2016). Panethnic differences in blood pressure in Europe: a systematic review and meta-analysis. *PloS one, 11*(1), e0147601.
